# Development of LC-MS/MS Database Based on 250 Potentially Highly Neuroactive Compounds and Their Metabolites

**DOI:** 10.3390/metabo15100650

**Published:** 2025-09-30

**Authors:** Taylor Teitelbaum, Haoduo Zhao, Lauren E. Koval, Yun-Chung Hsiao, Chih-Wei Liu, Julia E. Rager, Stephanie M. Engel, Kun Lu

**Affiliations:** 1 Department of Chemistry, University of North Carolina at Chapel Hill, Chapel Hill, NC 27599, USA; tteitel@ad.unc.edu; 2 Department of Environmental Sciences and Engineering, University of North Carolina at Chapel Hill, Chapel Hill, NC 27599, USA

**Keywords:** exposomics, neuroactive, mass spectrometry, database, metabolites

## Abstract

**Background:** Environmental chemicals are hypothesized to contribute to the development of neurodevelopmental disorders; however, only a fraction of the thousands of chemicals in common commercial use have validated assays. We recently developed the Environmental NeuRoactIve Chemicals (ENRICH) list of 250 chemicals prioritized for further testing due to their high likelihood of neuroactivity and human exposure, as derived through analysis across eight neuroactivity, exposure, and detection databases. Measuring some of these compounds in human biological media remains challenging due to the lack of information regarding their metabolites and detection frequencies. **Methods:** We created an LC-MS/MS database based on the targets in the ENRICH list using S9 human liver fractions to metabolize compounds individually and in groups into newly and previously discovered phase I metabolites. **Results:** The final database consisted of 274 compounds with 94 parent compounds and 182 metabolites being featured. A total of 55 novel metabolites were discovered. The confidence of the compounds, which were annotated correctly within the database, was high, increasing the odds of positive identifications within future exposomic work. The confidence of the annotations fell between the levels 1–3, with levels one and two consisting of 87% of the database. **Conclusions:** The creation of this database creates the opportunity for future biological studies centered around the impact these compounds and their metabolites have on the brain and for a better understanding of neurodevelopmental disorders and their origins.

## 1. Introduction

The origin of disorders and diseases is a questionable concern that has persisted throughout history. Prior research on molecular epidemiology has placed a strong emphasis on the identification of chronic diseases through gene expression, with a lack of focus on the environmental exposures that could cause the alterations of genes and disease formation [1]. Wild sought to challenge this knowledge gap by coining a term that encompassed the inter-relationship between genetics and the environment and their impact on humans: the exposome. The exposome describes all factors that impact a human throughout the entirety of their lifetime, including exogenous, endogenous, and lifestyle factors [2]. The concept of the exposome enhances the accuracy of disease origin by focusing simultaneously on environmental influences and their impact on the internal workings of the human body [3]. The implementation of the exposome into epidemiology studies is favorable but challenging due to the expansive number of chemicals that comprise it. With the chemical space alone being predicted to encompass over 1 × 10^61^ of compounds with a structure of less than 500 Da, it is an insurmountable feat to predict the full extent of the exposome’s chemical makeup [4]. Thus, a major bottleneck of exposomic analysis is the inability to annotate exposures properly. One field of study where this bottleneck is apparent is in the analysis of neurodevelopmental disorders.

As of 2024, only 1000 compounds have been validated to possess neurotoxicity via animal models [5]. The expansive exposome reveals there is a high probability that there are more unanalyzed chemicals that contribute towards neurotoxicity [4]. Recent studies have made efforts toward the identification of targets in the exposome with neurological potential. Utilizing neuroactivity, exposure, and detection databases, Rager et al. prioritized compounds likely to impact neurological systems, creating the Environmental NeuRoactIve Chemicals (ENRICH) list [6]. The purpose of the ENRICH list is to highlight compounds with a high potential to be neuroactive in order to screen for these compounds in human biological media. The list comprises 250 chemicals that have prior evidence of neuroactivity, are detectable in human samples, and are prevalent in products children may encounter. The ENRICH list effectively targets compounds with a greater need for neurotoxicity evaluations. To apply this list as targets in human biomonitoring studies, one must develop a technique that can analyze large quantities of compounds simultaneously, as well as target the vast chemical properties held by the 250 compounds and their metabolites. Mass spectrometry is a tool capable of performing such a task.

Advancements in analytical chemistry have led to the creation of HRMS, which has become a critical tool for identifying chemicals that may contribute to neurodevelopmental disorders. A non-targeted analysis (NTA) performed via HRMS can capture ~30,000–100,000 molecular features in biological samples, such as urine and blood [7,8,9,10,11]. For future human biomonitoring analyses, the use of HRMS provides an accessible means of detecting a copious number of compounds while providing a non-invasive means of analyzing children and their neural exposome through urine analysis. Thus, HRMS is an ideal tool for the measurement of ENRICH list targets and future targeted studies of these compounds in humans. Such analyses would be limited by the major bottleneck of compound annotation found in mass spectrometry and metabolomics [12]. The metabolome consists of all metabolites produced by the human body, and its size is not well understood due to the inability to know all the chemicals introduced to the human body and how they are metabolized [13]. Many metabolites, including those found in the ENRICH list, have not been evaluated and thus cannot yet be annotated by mass spectrometry. Our group addressed the gap in knowledge surrounding compound annotation and provided an accessible means to study the ENRICH list with respect to human neurodevelopment by creating an MS database centered around the ENRICH-listed compounds and their metabolites. The LC-MS/MS database was created by analyzing the detectable parent compounds within the ENRICH list and their metabolites created via S9 liver fraction experimentation. The results from this study will advance the field of environmental monitoring via exposomics as researchers continue to unravel complex relationships among the chemicals we are exposed to and their relationships to neural health outcomes.

## 2. Materials and Methods

### 2.1. Chemicals, Reagents, and Standards

All chemical standards that encompassed the ENRICH list compounds were purchased through Thermo Fisher Scientific (Rockford, IL, USA), Sigma Aldrich (Saint Louis, MO, USA), LGC Standards (Teddington, Middlesex, UK), Accustandard (New Haven, CT, USA), and Fluka (Seelze, Lower Saxony, Germany). Specific information regarding where each standard was purchased can be found in Table S1. The sodium phosphate monobasic monohydrate, sodium phosphate dibasic heptahydrate, magnesium chloride solution, DL-dithiothreitol, NADPH (tetrasodium salt), and human S9 liver fractions required for the S9 procedure were purchased from Sigma Aldrich (Saint Louis, MO, USA). Optimal LC-MS grade solvents (water, methanol, acetonitrile, acetone, and formic acid) were used and purchased from Thermo Fisher Scientific (Rockford, IL, USA). Individual target standards were prepared in concentrations ranging from 0.1 to 1 g/L. Parent Compound Mixtures (PCMs) were 10 µM standard mixtures of 10 different target compounds dissolved in 50% methanol. There were 25 PCMs prepared to account for the 250 chemical compounds on the ENIRCH list. The different compositions of all 25 PCMs, along with chemical IDs, are found in Table S1.

### 2.2. S9 Sample Preparation

**Figure 1:**
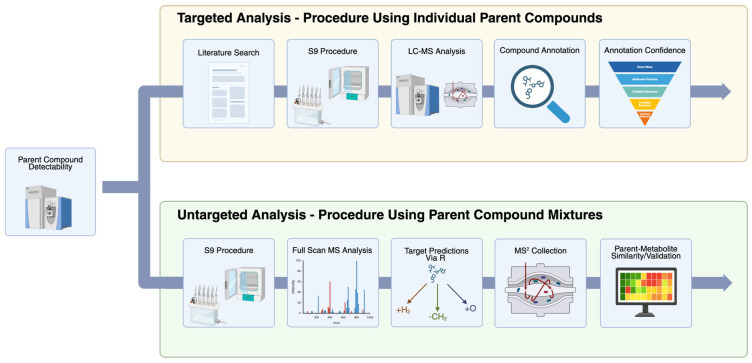
Overview of experimental methods for this study. Targeted and non-targeted analyses were performed to determine all detectable metabolites. The targeted analysis utilized the individual parent compound procedure, while the NTA utilized the parent compound mixture procedure.

An overview of all experimental proceedings is found in Figure 1. The following procedure was utilized in previous work with slight modifications [14,15,16]. The reaction was performed in a 50 mM phosphate buffer (pH = 7.4) comprising sodium phosphate monobasic monohydrate, sodium phosphate dibasic heptahydrate, and 10 mM of magnesium chloride. A 10 mM DL-dithiothreitol (DTT) solution and a 5 mM NADPH solution were then made utilizing the phosphate buffer. The microsome mixture was then prepared by diluting the S9 fractions to a concentration of 2 mg/mL with the DTT solution. The metabolic reaction was initiated by combining 40 µL of the target compound solution, 100 µL of the microsome mixture, and 60 µL of the NADPH solution. The methodology of the S9 procedures was validated through the individual analysis of propiconazole (CAS: 60207-90-1) and the detection of two of its reported metabolites in the literature [17]. The target compound solutions for the NTA were the 25 PCMs performed in replicates of 3 for each mixture, and the target compound solutions for the targeted analysis were the individual parent compounds at a concentration of 50 µM performed in a singular replicate. The reactions were incubated at 37 °C for two hours and stopped using 0.2 mL of cold acetone. The samples were centrifuged at 12,500 rpm for 10 min, and the supernatant was transferred to a new Eppendorf tube to remove any solid. The samples were then concentrated to complete dryness using a SpeedVac and reconstituted with 200 µL of 50% MeOH. The samples were centrifuged again at 12,500 rpm for 10 min, moved to glass vials, and were ready for MS analysis.

### 2.3. Mass Spectrometry Analysis

All parent compounds and their metabolites from the standard and S9 samples were analyzed via LC-MS/MS methods as described previously [18,19]. Two methods were performed for the instrumental analysis; one method type was a full scan during the non-targeted metabolite analysis, and the second method type was a parallel reaction monitoring (PRM) scan along with a full scan during the parent compound detectability and targeted metabolite analysis.

A Q Exactive Orbitrap mass spectrometer coupled to a Thermo Fisher Scientific Vanquish UHPLC (Thermo Fisher Scientific, Rockford, IL, USA) was utilized for both scans. The interface was a heated electrospray ionization source (HESI) with a positive ionization mode. Each prepared sample was injected (3 µL) into a Waters Acquity UPLC HSS T3 (reverse phase C_18_, 100 Å, 1.8 µm, 2.1 mm × 100 mm) analytical column with a controlled temperature of 40 °C. The mobile phases were water (A) and acetonitrile (B), both with 0.1% formic acid. The gradient of the elution was 15 min and as follows: 2% B from 0 to 1 min; 2% to 25% B from 1 to 3 min; 15% to 50% B from 3 to 6 min; 50% to 98% B from 6 to 7.5 min; 98% B held from 7.5 to 11.5 min; 98% to 2% B from 11.5 to 11.6 min; 2% held from 11.6 to 15 min for re-equilibration. The mass spectrometer was analyzed with the sheath gas, auxiliary gas, and sweep gas set to 50, 13, and 3, respectively. The HESI had a spray voltage of 3.50 kV, and the capillary and auxiliary heating temperatures were maintained at 263 and 425 °C, respectively. The full scan alone had a mass range from 70 to 1000 at a resolution of 70,000 fwhm (*m*/*z* 200) with an automatic gain control (AGC) of 3 × 10^6^ and a maximum injection time (MIT) of 225 ms. The scan that included PRM alongside a full scan had a resolution of 17,500 fwhm (*m*/*z* 200) with an isolation width of 1.2, an AGC of 3 × 10^5^, and an MIT of 100 ms.

### 2.4. Non-Targeted Metabolite Analysis

The discovery of metabolites not previously presented in the literature was based on an analysis of features with a mass-to-charge (*m*/*z*) ratio that corresponded to a predicted phase I metabolite of the target parent compounds. This portion of the experiment only focused on the parents successfully detected via the LC-MS/MS, as the predicted metabolite *m*/*z* values could not be assessed without the parent’s *m*/*z* value. Utilizing the S9 samples of the PCMs as previously mentioned, a full scan MS analysis was performed on all samples using the parameters established previously. The MS data were extracted via MS-Dial and further processed with R (v4.1.3, R Core Team, Vienna, Austria) and R Studio (v2024.04.0+735, R Studio Team, Boston, MA, USA) [20]. The code utilized known phase I metabolism transformations to predict the metabolites based on the parents, which was found in prior studies [21]. Features were only found to be significant if they were present in 50% of the samples, had a signal-to-noise ratio of at least 3, and, when compared to experimental blanks, had a fold-change greater than 2 and a *p*-value less than 0.05 via a paired sample t-test between the experimental blanks and the samples. A multiple test correction was not performed to increase the inclusivity of metabolites that could have been potentially relevant. Significant features were further filtered according to pre-established phase I metabolism rules. The rules established the different types of phase I reactions that could occur with resulting mass defects to the parent compounds; if a feature had an *m*/*z* ratio that matched the parent compound *m*/*z* ratio plus the mass defect, it was considered significant. The significant features were then targeted in an MS^2^ screening of the PCM S9 samples. The data was reprocessed with R and R Studio via the packages MSMSsim (1.0) and msentropy (ver. 0.1.4) to compare the MS^2^ spectrum similarities of the parent and its supposed metabolite [22,23]. The Similarity Between Two Mass Spectra functions (SpectrumSimilarity) compared the MS^2^ spectra of the parent and metabolite as vectors according to equations previously described and gave a similarity scoring between zero and one [24]. A score of zero reflected no similarities, while a score of one referred to identical similarities. A positive identity of the metabolite was made if it had a similarity score greater than or equal to 0.4; however, all metabolites with a score less than 0.4 were confirmed to be false positives or not through an MS^2^ analysis of the S9 sample for its corresponding parent compound alone.

### 2.5. Targeted Metabolite Analysis

This analysis highlighted metabolites discovered in prior literature and prediction software. The metabolites of all 250 compounds on the ENRICH list were researched, regardless of whether the parent compound could be detected via LC-MS/MS. This literature search was performed by searching “parent compound + metabolites” in Google and Google Scholar and/or utilizing the PubChem “Metabolism/Metabolites” section of the parent compound. BioTransformer (ver. 3.0)was the metabolite prediction software utilized to predict the human metabolites [25]. This web server was chosen due to its ability to focus on specific metabolic phases and utilize pre-established tools and machine learning to make predictions. The settings set in BioTransformer were as follows: a phase I transformation, combined CYP450 mode, and three reaction iterations. The phase I transformation parameters pertained to the phase I reaction performed during the S9 methodology; the combined CYP450 mode utilized rule-based and machine-learning techniques to predict the greatest number of metabolite possibilities; and the three reaction iterations expanded upon the number of potential metabolites without the assumption of the phase I metabolism only occurring once or twice. Once the known metabolites were identified, a tandem MS analysis of the S9 samples of the PCMs was performed to determine if a metabolite was present in a mixture containing its parent compound. If a signal was obtained, that same metabolite with its newly discovered retention time was evaluated for presence in the S9 sample containing only the parent compound via tandem mass spectrometry. All confirmed metabolites have been tentatively annotated according to their original sourcing of literature or BioTransformer. Confidence in the annotation was obtained by comparing the experimental spectra to online databases, including PubChem, mzCloud Advanced Mass Spectral Database, MassBank High Quality Mass Spectral Database (v2.2.8), Mass Frontier (v8.0-SR1), and CFM-ID (v4.0). All features with fragmentation patterns found in databases were assigned an annotation confidence of 2, and features with no fragmentation matches were assigned an annotation confidence of 3.

## 3. Results

### 3.1. S9 Method Validation

**Figure 2:**
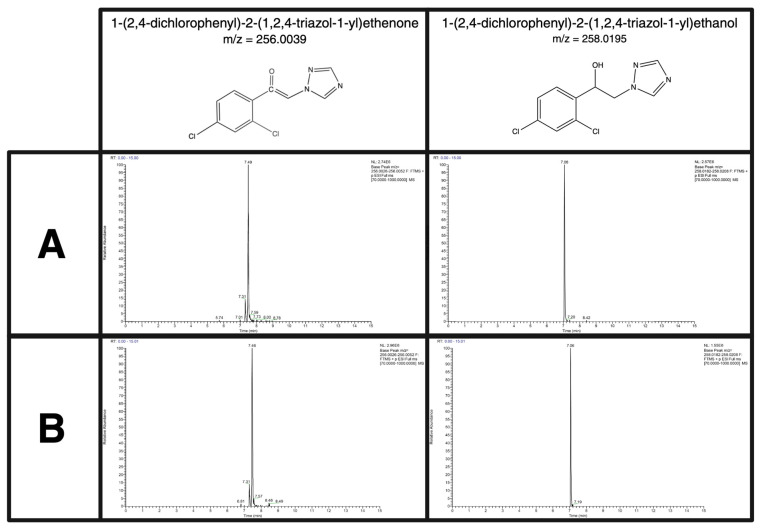
Spectral images of two known metabolites of the parent compound, propiconazole. Propiconazole has been reported in the literature to produce the metabolites 1-(2,4-dichlorophenyl)-2-(1,2,4-triazol-1-yl) ethenone and 1-(2,4-dichlorophenyl)-2-(1,2,4-triazol-1-yl) ethanol. Each respective metabolite is found above its own MS spectra. (**A**) Demonstrated the capability to produce the known metabolites when propiconazole underwent the S9 procedure alone. (**B**) Demonstrated the capability to produce the known metabolites when propiconazole underwent the S9 procedure in a compound mixture.

The experimental methodology of the S9 experimentation for both the non-targeted and targeted methods was validated through the examination of one parent compound, propiconazole, and its well-established metabolites. Propiconazole was first metabolized alone in the S9 mixture and found to produce two previously discovered metabolites, 1-(2,4-dichlorophenyl)-2-(1,2,4-triazol-1-yl) ethenone and 1-(2,4-dichlorophenyl)-2-(1,2,4-triazol-1-yl) ethanol as exemplified in Figure 2A. The database PubChem concurred with the annotation of the metabolites. The experiment was confirmed to successfully metabolize a 10-compound mixture by running the experiment using the PCM group, in which propiconazole was included. Figure 2B demonstrated the detectability of the same metabolites with similar normalization levels as the individual compound S9 experiment, demonstrating precision and accuracy with the procedure.

### 3.2. Parent Compound Detection

**Figure 3:**
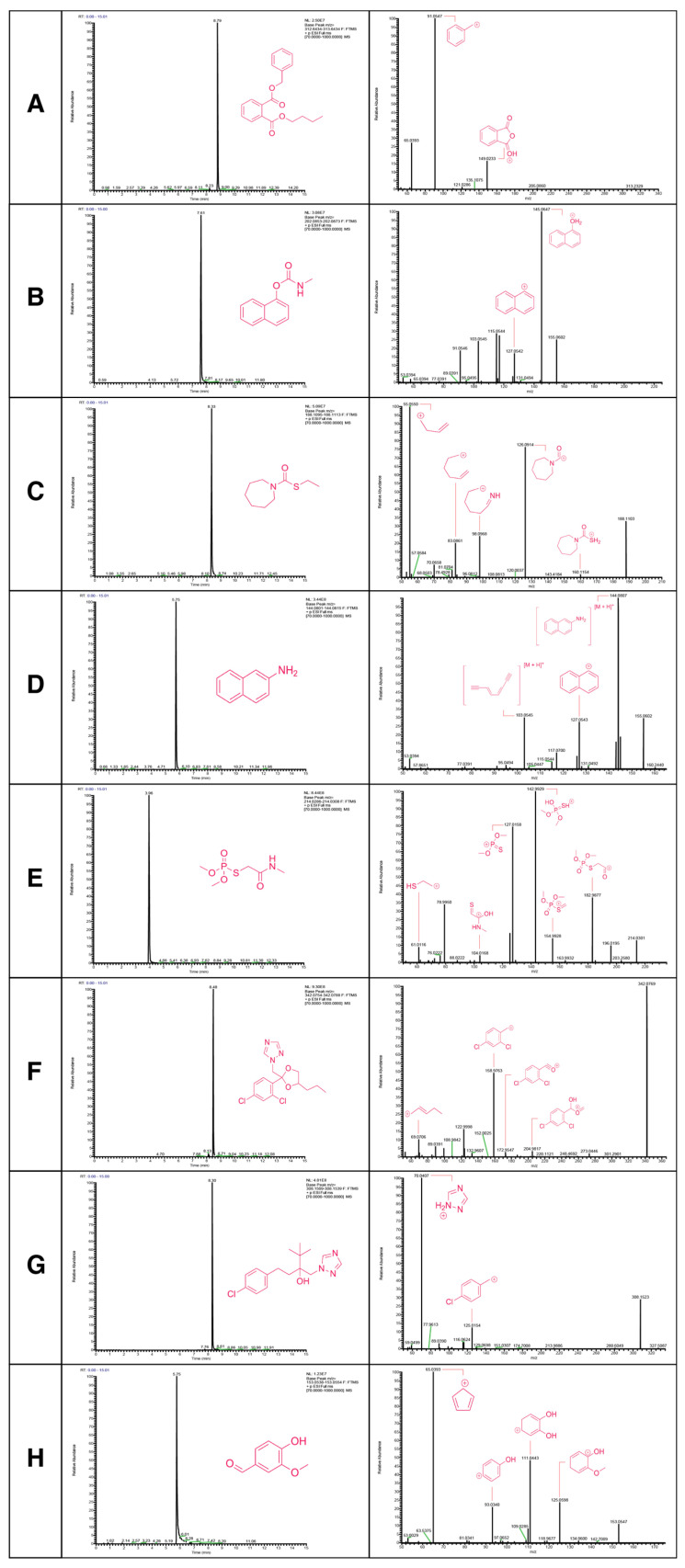
Chromatograms and MS^2^ spectra of eight example parent compounds detectable via LC-MS/MS. All eight parent compounds exemplified in this figure originated from different parent compound mixtures. The identity of the of each compound is as follows: (**A**) Benzyl butyl phthalate (CAS: 85-68-7); (**B**) Carbaryl (CAS: 63-25-2); (**C**) Molinate (CAS: 2212-67-1); (**D**) 2-Naphthylamine (CAS: 91-59-8); (**E**) Omethoate (CAS: 1113-02-6); (**F**) Propiconazole (CAS: 60207-90-1); (**G**) Tebuconazole (CAS: 107534-96-3); (**H**) Vanillin (CAS: 121-33-5).

To optimize the detectability of all 250 compounds featured on the ENRICH list, 25 compound mixtures consisting of 10 parent compounds each at a concentration of 10 µM were created. All parents were set as a target under the PRM analysis of the parent compound mixtures with an anticipated ionization of [M + H] for each target. Our detection capabilities are exemplified in Figure 3 through the chromatograms and MS^2^ spectra of eight of the several compounds we successfully measured. A total of 94 parent compounds were detected on the instrument utilizing a reversed-phase column with a positive ionization mode. The normalized abundance levels of all detectable chemicals ranged between 1 × 10^5^ and 1 × 10^9^. All detectable parent compounds have an annotation confidence of 1 due to the utilization of standards [26,27,28,29]. The detectable parent compounds and their MS identifiers are found in Table 1. 

### 3.3. Non-Targeted Metabolite Annotation

**Figure 4:**
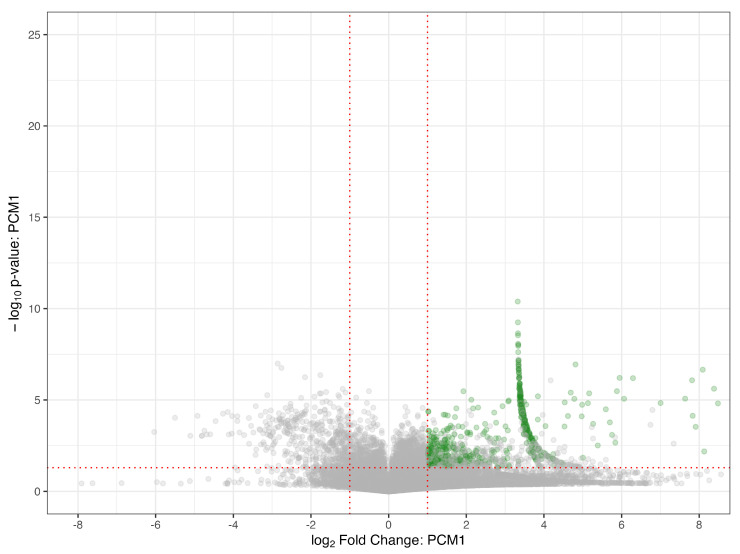
A volcano plot of the features found to be significant compared to experimental blanks in PCM 1 before the inclusion of the phase I metabolism rules. PCM 1 had a total of 568 significant features that were present in at least 50% of the samples, an S/N ratio of at least 3, a fold-change greater than or equal to 2, and a *p*-value less than 0.05 when compared to the experimental blanks.

**Figure 5:**
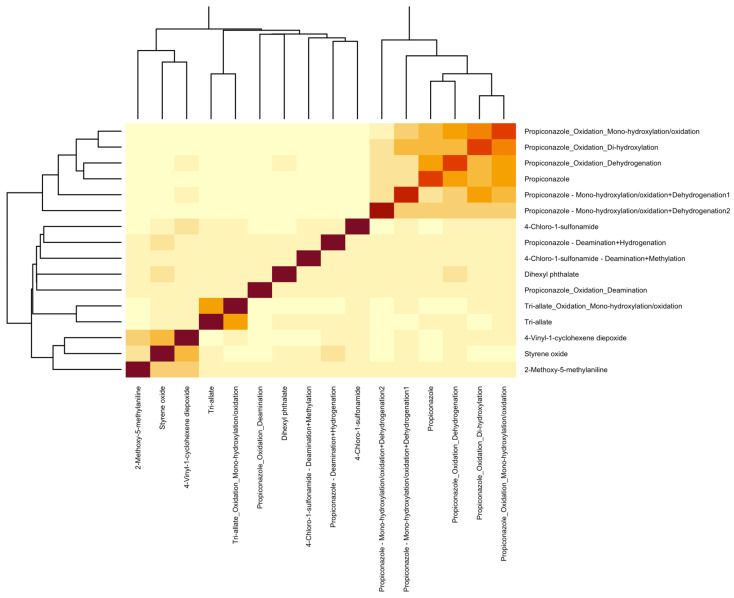
A heat map of the predicted metabolites and their parent compounds during the non-targeted metabolite analysis. The heat map was created by determining the MS/MS spectral entropy scores between the significant metabolites and the detectable parent compounds. Darker regions on the map signify a closer relation between the parent compounds and their metabolites.

**Figure 6:**
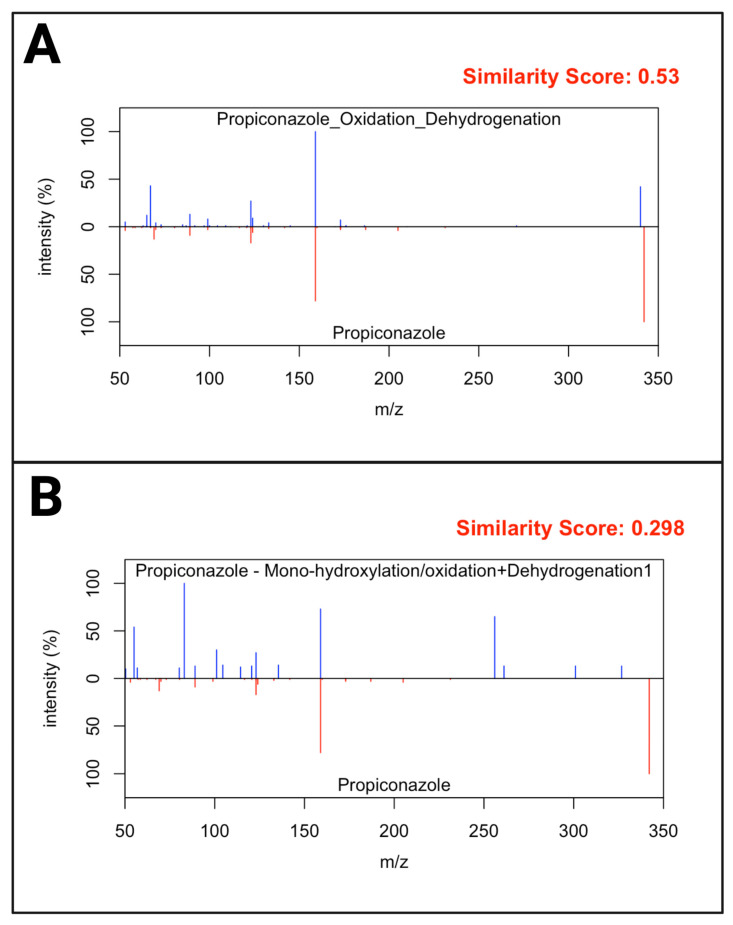
An MS^2^ comparison of propiconazole to two of its predicted metabolites during the NTA. (**A**) The spectral comparison represents a metabolite given a high similarity scoring due to the multitude of fragmentation matches to propiconazole and was automatically concurred to be a propiconazole metabolite. (**B**) The spectral comparison showcases a metabolite with a lower similarity score despite there being multiple fragmentation matches with its parent. This low scoring was attributed to the noise present in the metabolite MS^2^ spectrum, which is why all metabolites with a similarity score less than 0.4 were further evaluated.

The detection of and annotation of non-targeted metabolites was based on the usage of R scripting with pre-set phase I metabolism rules on full scan and PRM MS data. The full-scan MS data were first processed with the following parameters: present in 50% of the samples, had a signal-to-noise ratio of at least 3, had a fold-change greater than or equal to 2, and a *p*-value less than 0.05 when compared to the experimental blanks. The results of feature filtering based on these parameters are seen in Figure 4, which represents the filtering performed on PCM 1. On average, 537 features were significant following these parameters amongst all 25 PCM groupings. Those significant features were further processed to include only those that had a mass defect according to one of the phase I reaction rules. This step was limited to parent compounds with a detectability on the instrument due to the mass defect being calculated based on the original parent *m*/*z* value. The inclusion of the phase I metabolism rules filtered the significant feature to an average of 8 features per PCM group, with a minimum value of 0 features and a maximum value of 35 features. The MS^2^ of the potential metabolites and their parents were then evaluated for similarities, and this comparison was visualized in a heatmap for PCM 1 in Figure 5. A total of 16 metabolites were found to have a similarity score greater than 0.4 when compared to their parent. The remaining metabolites with similarity scores less than 0.4 were reevaluated using the individual parent compound S9 sample, and another 25 metabolites were confirmed and validated. This reevaluation was performed since metabolites do not always maintain a similar structure when compared to their parent, as well as to account for noise that lowers the similarity scoring, as witnessed in Figure 6. When compared to the results of known metabolites in the literature as well as metabolites predicted by BioTransformer, the R Script methodology successfully detected 24 novel metabolites. All novel metabolites were assigned an annotation confidence of 3 due to being unable to confirm the identity via a database, but having further confirmation through the similarity scorings or detection in the individual parent compound S9 sample. The detectable NTA metabolites and their MS identifiers are found in Table 1.

### 3.4. Targeted Metabolite Annotation

**Figure 7:**
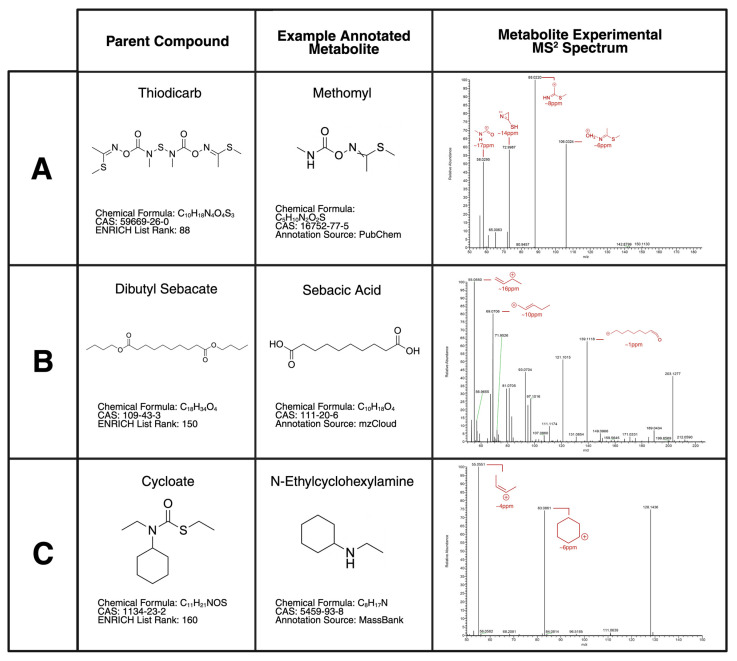
The annotation of metabolites previously found in the literature or predicted by BioTransformer via MS databases. The metabolites are presented alongside their parent compound of origin and the MS^2^ data obtained. The represented parent compounds include (**A**) Thiodicarb (CAS: 59669-26-0), (**B**) Dibutyl sebacate (CAS: 109-43-3), and (**C**) Cycloate (CAS: 1134-23-2). The MS^2^ spectra highlight the fragmentation of the metabolites as well as the ppm difference when compared to the MS databases.

The detection of and annotation of targeted metabolites was based on a manual literature search, along with the usage of BioTransformer. The combination of both metabolite sourcing yielded results for 201 parent compounds on the ENRICH list. The metabolites derived from these searches served as the targets for PRM in the analysis of the PCM and individual S9 samples. The analysis of the PCM S9 samples provided the statistical significance in the repeatable production of the target metabolites, while the analysis of the individual S9 samples provided the accuracy in assigning the metabolites to their specific parent compound. The PRM scan of all samples revealed a total of 161 metabolites detectable via the LC-MS/MS. Of the 161 metabolites, 128 originated from a literature source, while the remaining 31 were predicted by BioTransformer alone. The 31 BioTransformer metabolites were novel as well, having not been previously observed in any scientific journal. The verification of the metabolites’ identities was performed through a database comparison with either PubChem, mzCloud, MassBank, CFM-ID, or Mass Frontier. PubChem, mzCloud, and MassBank provided real-life data to make comparisons to, while CFM-ID and Mass Frontier provided predicted fragmentation patterns based on the compound given. A compound with information matching any of the databases received an annotation confidence of 2, while any other compound received an annotation confidence of 3. A sum of 149 out of the 161 metabolites had an annotation confidence of 2 assigned to them. The comparison of the fragments to the databases typically yielded differences in experimental and known data of less than 25 ppm, as demonstrated in Figure 7. The detectable targeted analysis metabolites and their MS identifiers are found in Table 1.

## 4. Discussion

This study sought to use S9 liver fractions in conjunction with mass spectrometry to develop a database of compounds and their metabolites that are highly potentially neuroactive. By performing S9 experimentation on individual compounds or groups of compounds within our recently published ENRICH list, we were able to successfully measure both previously discovered and novel metabolites from a copious number of chemicals [6]. The database centered around the detection of compounds via LC-MS/MS instrumentation with an ESI source in positive mode and a C18 column to perform reversed-phase liquid chromatography. The final database consisted of a total of 274 compounds, as shown in Table 1. Detailed information, such as specific fragmentation and methods of annotation, can be found in Table S2. Altogether, 94 parent compounds and 182 metabolites were detected via our methods. Two of the parent compounds were also found to be metabolites; therefore, the duplicated compounds were condensed into one annotation. While the size of the database is a significant feat, it is also important to note the high confidence found within the database.

Compound annotation is one of the most critical and difficult aspects in the world of metabolomics and exposomics [30,31]. Despite the difficulty being highly attributed to the lack of annotated substances, the challenge of compound annotation is further heightened through annotation confidence. The Metabolomics Standards Initiative (MSI) proposed the idea of annotation confidence with metabolomic MS data to standardize data for all scientists to easily utilize and replicate within their own studies [27]. The confidence levels for compound annotation range from one to five, with one being the highest level of confidence. The higher level of confidence one obtains while annotating, the more reliable their results and conclusions are in their metabolomic or exposomic studies. Thus, the ideal confidences for MS databases are a level two, which refers to a probable structure, and a level one, which refers to a confirmed structure [26,27,28,29]. We sought to build a database to match these set standards and were successful in obtaining most of the confidence levels for our annotation at levels one or two. While we do have some metabolites with a level 3 annotation or a tentative structure, these annotations only account for 13% of the database and can be improved through additional analytical analyses, such as NMR.

Through the creation of an MS database with high confidence levels, we aided the knowledge gap for various scientific areas. The first targeted scientific area was compound annotation within mass spectrometry. As previously mentioned, compound annotation is greatly limited by the number of MS databases present as well as the lack of annotation confidence [30,31].

The lack of annotation resources is heavily attributed to the inability to predict and produce standards for the predicted 1,000,000 chemicals that encompass the metabolome and the unpredictable number of chemicals that encompass the exposome [4,32]. There have been significant efforts to reduce this bottleneck through database mining and fragmentation predictions; however, real-life data is the true indicator for the vastly unpredictable metabolome and exposome [29,30,31,33,34]. Thus, our experimental MS data with higher levels of confidence can initiate further steps towards closing the bottleneck of mass spectrometry analyses.

Along with mass spectrometry, metabolome discovery knowledge is enhanced through the publication of this study. The discovery of new metabolites has been a slow and monotonous process due to the bulk of literature focusing on the metabolism of one or a few compounds at a time [35,36,37]. This fact could be true based on the increase in difficulty identifying the origins of a metabolite as the number of parent compounds studied simultaneously increases. The methodology utilized in this article challenges the standard of metabolite discovery through the simultaneous qualification of metabolites from 10 parent compounds. This mass metabolite annotation and discovery proved effective in the recording of 182 detectable metabolites. Among the 182 detectable metabolites, 55 were discovered to be novel and had never been reported in the literature before. Expanding upon the number of compounds analyzed after undergoing phase I metabolism experimentation increased the rates of metabolite discovery and could be an essential tool in furthering the knowledge base on the metabolome in a faster, cost-effective manner.

The most significant contribution to the development of the MS database provided, and the drive of this paper’s publication, was the area of neurodevelopment. To effectively evaluate the risks of the chemicals with a high potential of neuroactivity proposed from the ENRICH list, one must possess the capabilities to measure the compound within biological samples [6]. The neuroactive chemicals MS database enables this evaluation to become a reality. By applying this newly founded MS data to biological experiments, specifically to early-life studies, the field of neurodevelopment will be expanded upon with the enhanced knowledge of the neurotoxicity of more chemicals.

There are limitations to be considered with this experimental design despite the significant scientific contribution. One limiting factor was the inability to detect all target parent compounds and their metabolites via LC-MS/MS. The ENRICH list included compounds that could be detected via GC-MS and/or LC-MS; however, many of the targets were preferentially detected via GC-MS [6]. This setback can be alleviated through the repeated analyses of all the samples created during this experimental procedure, except on a GC-MS/MS instead of an LC-MS/MS. Another limitation associated with the methodology is the biases found with the MS analysis. For both the full scan and PRM scan of all samples, the target chemicals were separated via reversed-phase liquid chromatography and ionized in a positive mode. While previous studies produced from our lab have proven that positive ionization in combination with reversed-phase liquid chromatography yields the greatest number of results and detection, they fail to account for polar parents and metabolites with higher ionization potential in a negative mode [38,39,40]. Given more time, this limitation could be resolved with the analysis of all parents and their metabolites in both positive and negative ionization modes and with both normal-phase and reversed-phase liquid chromatography. Regardless of the limitations, our experimental methodology effectively created an LC-MS/MS based on chemicals with high neuroactive potential to contribute towards the future of neurodevelopmental studies.

## 5. Conclusions

Neurodevelopment within a child going through early-life stages is critical and easily impacted by the exponential growth and plasticity of the child’s mind. However, the causations of neurodevelopmental impairment, particularly with respect to chemical exposure, are widely not understood due to understudied chemicals within the vast human exposome. We desired to fill in exposomics and neurodevelopmental knowledge gaps by constructing a tailorable LC-MS/MS database based on the ENRICH list and its 250 compounds with a high potential of neuroactivity. This goal was accomplished through the phase I metabolism of all 250 targets via human S9 liver fractions in a targeted and non-targeted analysis. The resulting MS list contained 274 compounds, represented by 94 parent compounds and 182 metabolites. Of the 274 annotated chemicals, 55 metabolites were newly discovered. This database enables the discovery of more xenobiotic compounds found within a biological subject’s body and links them directly to neurodevelopmental changes. To further aid neurodevelopmental analyses and identify the optimal location within the body to detect potentially neuroactive targets, our group seeks to perform a future in vivo study on the detectability and pharmacokinetics of the chemicals featured in the database.

## Figures and Tables

**Table 1:** LC-MS/MS Database of Compounds with a High Neuroactive Potential and their Metabolites.

Chemical Name	Formula	Compound Type	Mass-to-Charge Ratio	Retention Time (min)
1-((4-allyl-2-(2,4-dichlorophenyl)-1,3-dioxolan-2-yl)methyl)-1H-1,2,4-triazole	C_15_H_15_Cl_2_N_3_O_2_	Metabolite	340.0614	8.31
1-(2,4-dichlorophenyl)-2-(1,2,4-triazol-1-yl)ethanol	C_10_H_9_Cl_2_N_3_O	Metabolite	258.0195	7.07
1-(2,4-Dichlorophenyl)-2-(1h-1,2,4-triazol-1-yl)ethanone	C_10_H_7_Cl_2_N_3_O	Metabolite	256.0039	7.32
1-(4-Chlorophenyl)-4,4-dimethyl-3-(1,2,4-triazol-1-ylmethyl)pent-1-en-3-ol	C_16_H_20_ClN_3_O	Metabolite	306.1368	8.28
1-(4-Hydroxyphenyl)-1-nonanone	C_15_H_22_O_2_	Metabolite	235.1693	8.77
1-(6-(hydroxymethyl)-2-(phenylamino)pyrimidin-4-yl)cyclopropan-1-ol	C_14_H_15_N_3_O_2_	Metabolite	258.1237	5.75
1-Acenaphthenone	C_12_H_8_O	Metabolite	169.0648	7.25
1-Acenaphthenone	C_12_H_8_O	Metabolite	169.0648	8.02
1-Amino-2-naphthol-6-sulfonic acid	C_10_H_9_NO_4_S	Metabolite	240.0325	1.07
1-Amino-2-naphthol-6-sulfonic acid	C_10_H_9_NO_4_S	Metabolite	240.0325	3.63
1-Aminonaphthalene	C_10_H_9_N	Parent	144.0808	6.25
1-Naphthyl (hydroxymethyl)carbamate	C_12_H_11_NO_3_	Metabolite	218.0812	5.02
1-Naphthyl (hydroxymethyl)carbamate	C_12_H_11_NO_3_	Metabolite	218.0812	6.71
1-Naphthyl carbamate	C_11_H_9_NO_2_	Metabolite	188.0706	4.31
1,2-Benzisothiazol-3(2H)-one	C_7_H_5_NOS	Parent	152.0165	5.61
1,2-benzisothiazol-3(2H)-one 1-oxide	C_7_H_5_NO_2_S	Metabolite	168.0114	5.37
1,2-Benzisothiazol-3(2H)-one_Reduction_Hydrogenation	C_7_H_7_NOS	Metabolite	154.0319	5.412
1,2-Diacetylbenzene	C_10_H_10_O_2_	Parent	163.0754	6.44
1,2-Diacetylbenzene_Reduction_Dehydroxylation/decarboxylation	C_10_H_10_O	Metabolite	147.0804	7.608
1,2,3-Benzothiadiazole-7-carboxylic acid	C_7_H_4_N_2_O_2_S	Metabolite	181.0066	6.36
1,3-Diphenylguanidine	C_13_H_13_N_3_	Parent	212.1182	5.36
1,3-Diphenylguanidine - Deamination + Dehydroxylation/decarboxylation	C_13_H_10_N_2_	Metabolite	195.0915	5.47
1,3-Diphenylguanidine - Deamination + Hydrogenation	C_13_H_12_N_2_O	Metabolite	213.1020	7.797
1,3,5-Triazine-2,4-diamine, 6-chloro-n-ethyl-n’-hydroxy-	C_5_H_8_ClN_5_O	Metabolite	190.0490	8.94
1[[2(2,4-dichlorophenyl)-4-hydroxypropyl-1,3-dioxolane-2-yl]methyl]1h-1,2,4triazole	C_15_H_17_Cl_2_N_3_O_3_	Metabolite	358.0720	7.49
2-(3,5-Dichlorophenylcarbamoyl)-1,2-dimethylcyclopropane-1-carboxylic acid	C_13_H_13_Cl_2_NO_3_	Metabolite	302.0345	5.16
2-(4-Oxopentoxycarbonyl)benzoic acid	C_13_H_14_O_5_	Metabolite	251.0914	3.38
2-(5-Carboxypentoxycarbonyl)benzoic acid	C_14_H_16_O_6_	Metabolite	281.1020	7.96
2-(Phenylazo)phenol	C_12_H_10_N_2_O	Metabolite	199.0866	7.43
2-(Phenylazo)phenol	C_12_H_10_N_2_O	Metabolite	199.0866	8.18
2-Anilino-6-cyclopropylpyrimidine-4-carbaldehyde	C_14_H_13_N_3_O	Metabolite	240.1131	6
2-Anthrol	C_14_H_10_O	Metabolite	195.0804	7.09
2-Butanone oxime	C_4_H_9_NO	Parent	88.0757	4.6
2-Ethyl-6-oxohexyl diphenyl phosphate	C_20_H_25_O_5_P	Metabolite	377.1512	8.59
2-ethylhex-5-en-1-yl bis(2-ethylhexyl) phosphate	C_24_H_49_O_4_P	Metabolite	433.3441	12.22
2-ethylhex-5-en-1-yl bis(2-ethylhexyl) phosphate	C_24_H_49_O_4_P	Metabolite	433.3441	12.47
2-ethylhex-5-en-1-yl diphenyl phosphate	C_20_H_25_O_4_P	Metabolite	361.1563	9.03
2-Ethylhexyl diphenyl phosphate	C_20_H_27_O_4_P	Parent	363.1720	9.27
2-Hydroxy-desmethyldiuron	C_8_H_8_Cl_2_N_2_O_2_	Metabolite	235.0036	7.68
2-hydroxy-N-methylsuccinimide	C_5_H_7_NO_3_	Metabolite	130.0499	0.68
2-hydroxy-N-methylsuccinimide	C_5_H_7_NO_3_	Metabolite	130.0499	1.17
2-hydroxy-N-methylsuccinimide	C_5_H_7_NO_3_	Metabolite	130.0499	1.23
2-Mercaptobenzo[d]thiazol-6-ol	C_7_H_5_NOS_2_	Metabolite	183.9885	7.22
2-Mercaptobenzothiazole	C_7_H_5_NS_2_	Metabolite	167.9936	7.23
2-Methoxy-5-methylaniline	C_8_H_11_NO	Parent	138.0913	4.55
2-N-butan-2-yl-5-tert-butyl-3-nitrobenzene-1,2-diamine	C_14_H_23_N_3_O_2_	Metabolite	266.1863	8.94
2-n-Octyl-4-isothiazolin-3-one	C_11_H_19_NOS	Parent	214.1260	8.18
2-n-Octyl-4-isothiazolin-3-one -Hydrogenation + Epoxide-hydration	C_11_H_23_NO_2_S	Metabolite	234.1518	6.486
2-n-Octyl-4-isothiazolin-3-one -Mono-hydroxylation/oxidation + Epoxide-hydration	C_11_H_21_NO_3_S	Metabolite	248.1311	6.409
2-Naphthol	C_10_H_8_O	Metabolite	145.0648	5.72
2-Naphthylamine	C_10_H_9_N	Parent	144.0808	5.75
2-Nitrobutane	C_4_H_9_NO_2_	Metabolite	104.0706	0.66
2-Octyl-1H-1lambda~4~,2-thiazole-1,3(2H)-dione	C_11_H_19_NO_2_S	Metabolite	230.1209	3.07
2-Octyl-1H-1lambda~4~,2-thiazole-1,3(2H)-dione	C_11_H_19_NO_2_S	Metabolite	230.1209	6.43
2-Octyl-1H-1lambda~4~,2-thiazole-1,3(2H)-dione	C_11_H_19_NO_2_S	Metabolite	230.1209	6.7
2-Tert-butyl-4-ethenylphenol	C_12_H_16_O	Metabolite	177.1274	7.61
2-Tert-butyl-4-ethenylphenol	C_12_H_16_O	Metabolite	177.1274	7.98
2-Tert-butyl-4-ethenylphenol	C_12_H_16_O	Metabolite	177.1274	8.02
2,3-Dihydroxypropanamide	C_3_H_7_NO_3_	Metabolite	106.0499	0.66
2,4-Diaminophenol	C_6_H_8_N_2_O	Metabolite	125.0709	0.76
2,4-Diaminophenol	C_6_H_8_N_2_O	Metabolite	125.0709	1.17
2,5-Di-tert-butylhydroquinone	C_14_H_22_O_2_	Parent	223.1693	9.16
2,5-Hexanedione	C_6_H_10_O_2_	Metabolite	115.0754	5.23
2,6-Diethylaniline	C_10_H_15_N	Parent	150.1277	7.68
3-((1H-1,2,4-triazol-1-yl)methyl)-5-(4-chlorophenyl)-3-hydroxy-2,2-dimethylpentanoic acid	C_16_H_20_ClN_3_O_3_	Metabolite	338.1266	7.63
3-Acetamidobenzoic acid	C_9_H_9_NO_3_	Metabolite	180.0655	5.13
3-Amino-7-hydroxy-3,4-dihydrochromen-2-one	C_9_H_9_NO_3_	Metabolite	180.0655	5.13
3-Iodo-2-propynyl-N-butylcarbamate	C_8_H_12_INO_2_	Parent	281.9985	7.97
4-(But-2-en-2-yl)phenol	C_10_H_12_O	Metabolite	149.0961	6.25
4-(But-2-en-2-yl)phenol	C_10_H_12_O	Metabolite	149.0961	7.3
4-(Ethylamino)phenol	C_8_H_11_NO	Metabolite	138.0913	2.22
4-(non-1-en-1-yl)phenol	C_15_H_22_O	Metabolite	219.1743	8.1
4-chloro-2-cyano-5-(4-(hydroxymethyl)phenyl)N,N-dimethyl-1h-imidazole-1-sulfonamide	C_13_H_13_ClN_4_O_3_S	Metabolite	341.0470	7.78
4-Chloro-2-cyano-N,N-dimethyl-5-(4-methylphenyl)-1H-imidazole-1-sulfonamide	C_13_H_13_ClN_4_O_2_S	Parent	325.0521	8.49
4-Chloro-2-cyano-N,N-dimethyl-5-(4-methylphenyl)-1H-imidazole-1-sulfonamide - Deamination + Methylation	C_14_H_12_ClN_3_O_3_S	Metabolite	338.0356	6.691
4-Chloro-5-(4-(hydroxymethyl)phenyl)-imidazole-2-carbonitrile	C_11_H_8_ClN_3_O	Metabolite	234.0429	6.89
4-Chloroaniline	C_6_H_6_ClN	Parent	128.0262	4.86
4-Hept-6-enylphenol	C_13_H_18_O	Metabolite	191.1430	7.94
4-Hept-6-enylphenol	C_13_H_18_O	Metabolite	191.1430	8.23
4-Hydroxyazepan-2-one	C_6_H_11_NO_2_	Metabolite	130.0863	0.57
4-Hydroxychlorpropham	C_10_H_12_ClNO_3_	Metabolite	230.0578	7.39
4-Hydroxychlorpropham	C_10_H_12_ClNO_3_	Metabolite	230.0578	7.47
4-Hydroxydiphenylamine	C_12_H_11_NO	Metabolite	186.0913	7.62
4-Methylimidazole	C_4_H_6_N_2_	Parent	83.0604	0.74
4-Octanoylphenol	C_14_H_20_O_2_	Metabolite	221.1536	8.93
4-Vinyl-1-cyclohexene diepoxide	C_8_H_12_O_2_	Parent	141.0910	5.56
4,4_-Methylene-bis(2-methylaniline)_Oxidation_Dehydrogenation	C_15_H_16_N_2_	Metabolite	225.1392	4.56
4,4_-Methylene-bis(2-methylaniline)_Oxidation_Dehydrogenation	C_15_H_16_N_2_	Metabolite	225.1392	4.78
4,4_-Methylene-bis(2-methylaniline)_Reduction_Methylation	C_16_H_20_N_2_	Metabolite	241.1697	5.011
4,4′-Methylene-bis(2-methylaniline)	C_15_H_18_N_2_	Parent	227.1543	4.67
5-(4-Chlorophenyl)-2,2-dimethyl-3-(1H-1,2,4-triazol-1-ylmethyl)-1,3-pentanediol	C_16_H_22_ClN_3_O_2_	Metabolite	324.1473	7.74
5-[[2-(2-Ethylhexoxycarbonyl)benzoyl]oxymethyl]heptanoic acid	C_24_H_36_O_6_	Metabolite	421.2585	5.91
5-HO-Ehdpp	C_20_H_27_O_5_P	Metabolite	379.1669	8.38
5-HO-Ehdpp	C_20_H_27_O_5_P	Metabolite	379.1669	8.48
5-Hydroxy-1-methylpyrrolidin-2-one	C_5_H_9_NO_2_	Metabolite	116.0706	0.73
5,5′-Dimethoxy-3,3′-di-tert.-butyl-1,1′-biphenyl-2,2′-diol	C_22_H_30_O_4_	Metabolite	359.2217	6.74
5,5′-Dimethoxy-3,3′-di-tert.-butyl-1,1′-biphenyl-2,2′-diol	C_22_H_30_O_4_	Metabolite	359.2217	6.83
6-Butoxy-6-oxohexanoic acid	C_10_H_18_O_4_	Metabolite	203.1278	7.51
6-Methylquinoline	C_10_H_9_N	Parent	144.0808	4.62
7,12-Benz(a)anthraquinone	C_18_H_10_O_2_	Metabolite	259.0753	8.23
7,12-Benz(a)anthraquinone	C_18_H_10_O_2_	Metabolite	259.0753	8.44
8-Quinolinol	C_9_H_7_NO	Parent	146.0600	3.66
9-(Oxiran-2-yl)nonanoic acid	C_11_H_20_O_3_	Metabolite	201.1485	6.22
Acedoben	C_9_H_9_NO_3_	Metabolite	180.0655	5.14
Acenaphthylene oxide	C_12_H_8_O	Metabolite	169.0648	6.13
Acetamide, N-(2-methoxyphenyl)-	C_9_H_11_NO_2_	Metabolite	166.0863	3.29
Acibenzolar-S-methyl	C_8_H_6_N_2_OS_2_	Parent	210.9994	8.23
Acibenzolar-S-methyl_Oxidation_Desulphuration	C_8_H_6_N_2_O_2_S	Metabolite	195.0222	7.798
Amitraz	C_19_H_23_N_3_	Parent	294.1965	8.99
Aniline, 4-tert-butyl-2,6-dinitro-	C_10_H_13_N_3_O_4_	Metabolite	240.0979	9.15
Azobenzene	C_12_H_10_N_2_	Parent	183.0917	8.09
Bensulide	C_14_H_24_NO_4_PS_3_	Parent	398.0678	8.56
Bensulide oxon	C_14_H_24_NO_5_PS_2_	Metabolite	382.0906	7.94
Benzaldehyde	C_7_H_6_O	Metabolite	107.0491	3.27
Benzidine	C_12_H_12_N_2_	Metabolite	185.1073	8.2
benzo [8,9]tetrapheno [1,2-b]oxirene	C_22_H_12_O	Metabolite	293.0961	0.73
Benzo[a]anthracene-3,4-diol	C_18_H_12_O_2_	Metabolite	261.0910	6.83
Benzyl butyl phthalate	C_19_H_20_O_4_	Parent	313.1434	8.8
Bis(2-ethylhexyl) adipate	C_22_H_42_O_4_	Parent	371.3156	11.12
Bis(2-ethylhexyl) phosphate	C_16_H_35_O_4_P	Metabolite	323.2346	10.15
Bis(2-ethylhexyl) phthalate	C_24_H_38_O_4_	Parent	391.2843	11.06
but-3-en-1-yl dibutyl phosphate	C_12_H_25_O_4_P	Metabolite	265.1563	8.52
Butyl bis(3-hydroxybutyl) phosphate	C_12_H_27_O_6_P	Metabolite	299.1618	6.43
Butyl bis(3-hydroxybutyl) phosphate	C_12_H_27_O_6_P	Metabolite	299.1618	6.56
Butyl bis(3-hydroxybutyl) phosphate	C_12_H_27_O_6_P	Metabolite	299.1618	6.77
Butyl bis(3-hydroxybutyl) phosphate	C_12_H_27_O_6_P	Metabolite	299.1618	7.15
Butyl bis(3-hydroxybutyl) phosphate	C_12_H_27_O_6_P	Metabolite	299.1618	7.32
Butyl dihydrogen phosphate	C_4_H_11_O_4_P	Metabolite	155.0468	7.7
Butyl dihydrogen phosphate	C_4_H_11_O_4_P	Metabolite	155.0468	7.85
Butylate	C_11_H_23_NOS	Parent	218.1573	9.02
Butylparaben	C_11_H_14_O_3_	Parent	195.1016	7.99
Caprolactam	C_6_H_11_NO	Parent/Metabolite	114.0913	4.46
Carbaryl	C_12_H_11_NO_2_	Parent	202.0863	7.61
Chlorpropham	C_10_H_12_ClNO_2_	Parent	214.0629	8.33
Chromone	C_9_H_6_O_2_	Metabolite	147.0441	1.49
Coumarin	C_9_H_6_O_2_	Parent	147.0441	6.76
Coumarin - Methylation + Epoxide-hydration	C_10_H_10_O_3_	Metabolite	179.0700	6.825
Cycloate	C_11_H_21_NOS	Parent	216.1417	8.88
Cycloate_Oxidation_Mono-hydroxylation/oxidation	C_11_H_21_NO_2_S	Metabolite	232.1363	7.268
Cyclohexanone Oxime	C_6_H_11_NO	Metabolite	114.0913	4.58
Cyclohexyl phenyl ketone	C_13_H_16_O	Parent	189.1274	8.7
Cyclohexylamine	C_6_H_13_N	Parent	100.1121	2.36
Cyprodinil	C_14_H_15_N_3_	Parent	226.1339	7.97
D-Sorbitol	C_6_H_14_O_6_	Parent	183.0863	0.66
DEET	C_12_H_17_NO	Parent	192.1383	7.72
DEET_Oxidation_Mono-hydroxylation/oxidation	C_12_H_17_NO_2_	Metabolite	208.1331	6.175
DEET_Oxidation_N/O-Dealkylation/demethylation	C_11_H_15_NO	Metabolite	178.1226	7.257
DEET_Reduction_Methylation	C_13_H_19_NO	Metabolite	206.1538	7.911
Deisopropyl Atrazine	C_5_H_8_ClN_5_	Parent	174.0541	5.28
Di-(2-Ethylhexyl) (2-Ethyl-6-Hydroxyhexyl) Phosphate	C_24_H_51_O_5_P	Metabolite	451.3547	10.27
Di-(2-Ethylhexyl) (2-Ethyl-6-Hydroxyhexyl) Phosphate	C_24_H_51_O_5_P	Metabolite	451.3547	10.53
Di-(2-Ethylhexyl) (2-Ethyl-6-Hydroxyhexyl) Phosphate	C_24_H_51_O_5_P	Metabolite	451.3547	10.77
Di-(2-Ethylhexyl) (2-Ethyl-6-Hydroxyhexyl) Phosphate	C_24_H_51_O_5_P	Metabolite	451.3547	10.91
Di-(2-Ethylhexyl) (2-Ethyl-6-Hydroxyhexyl) Phosphate	C_24_H_51_O_5_P	Metabolite	451.3547	10.98
Di-n-octyl phthalate	C_24_H_38_O_4_	Parent	391.2843	11.26
Diallyl phthalate	C_14_H_14_O_4_	Parent	247.0965	8.29
Diamyl Phthalate	C_18_H_26_O_4_	Parent	307.1904	9.25
Dibutyl 3-hydroxybutyl phosphate	C_12_H_27_O_5_P	Metabolite	283.1669	7.82
Dibutyl 3-hydroxybutyl phosphate	C_12_H_27_O_5_P	Metabolite	283.1669	7.99
Dibutyl adipate	C_14_H_26_O_4_	Parent	259.1904	8.86
Dibutyl phosphate	C_8_H_19_O_4_P	Metabolite	211.1094	7.73
Dibutyl phosphate	C_8_H_19_O_4_P	Metabolite	211.1094	8.53
Dibutyl phosphate	C_8_H_19_O_4_P	Metabolite	211.1094	8.7
Dibutyl Sebacate	C_18_H_34_O_4_	Parent	315.2530	9.62
Diethyl Succinate	C_8_H_14_O_4_	Parent	175.0965	7.23
Diethylene glycol	C_4_H_10_O_3_	Parent	107.0703	1.15
Diethylene glycol dimethyl ether	C_6_H_14_O_3_	Parent	135.1016	4.56
Diethylene Glycol Monobutyl Ether	C_8_H_18_O_3_	Parent	163.1329	6.21
Diethylene Glycol Monoethyl Ether	C_6_H_14_O_3_	Parent	135.1016	4.24
Diethylene glycol monomethyl ether	C_5_H_12_O_3_	Parent	121.0859	3.06
Diethylquinoneimine	C_10_H_13_NO	Metabolite	164.1070	3.88
Dihexyl phthalate	C_20_H_30_O_4_	Parent	335.2217	9.73
Diisobutyl adipate	C_14_H_26_O_4_	Parent	259.1904	8.83
Diisobutyl phthalate	C_16_H_22_O_4_	Parent	279.1591	8.88
Dimethyl glutarate	C_7_H_12_O_4_	Parent	161.0808	6.24
Dimethyl succinate	C_6_H_10_O_4_	Parent	147.0652	5.47
Dimethyl sulfoxide	C_2_H_6_OS	Parent	79.0212	0.74
Diphenylamine	C_12_H_11_N	Parent	170.0964	8.40
Diphenylamine - Mono-hydroxylation/oxidation + Dehydrogenation	C_12_H_9_NO	Metabolite	184.0757	7.689
Diuron	C_9_H_10_Cl_2_N_2_O	Parent	233.0243	7.74
Ethoprophos	C_8_H_19_O_2_PS_2_	Parent	243.0637	8.32
Ethoprophos - N/O-Dealkylation/demethylation + Desulphuration	C_7_H_17_O_3_PS	Metabolite	213.0707	7.661
Ethoprophos_Oxidation_N/O-Dealkylation/demethylation	C_7_H_17_O_2_PS_2_	Metabolite	229.0479	8.086
Ethyl Dihydrogen Phosphate	C_2_H_7_O_4_P	Metabolite	127.0155	5.18
Flumioxazin	C_19_H_15_FN_2_O_4_	Parent	355.1089	8.08
Flumioxazin_Reduction_Hydrogenation	C_19_H_17_FN_2_O_4_	Metabolite	357.1243	7.347
Formamide, N,N-bis(2-methylpropyl)-1-(ethylsulfinyl)-	C_11_H_23_NO_2_S	Metabolite	234.1522	8.07
Formamide, N,N-bis(2-methylpropyl)-1-(ethylsulfinyl)-	C_11_H_23_NO_2_S	Metabolite	234.1522	8.15
Gallic Acid	C_7_H_6_O_5_	Metabolite	171.0288	6.42
Hexamethylenetetramine	C_6_H_12_N_4_	Parent	141.1135	0.64
Isophorone	C_9_H_14_O	Parent	139.1117	7.39
Linuron	C_9_H_10_Cl_2_N_2_O_2_	Parent	249.0192	8.13
m-Nitroaniline	C_6_H_6_N_2_O_2_	Metabolite	139.0502	6.48
m-Toluidine	C_7_H_9_N	Parent	108.0808	3.37
Malaoxon	C_10_H_19_O_7_PS	Parent	315.0662	7.39
Malaoxon_Oxidation_N/O-Dealkylation/demethylation	C_9_H_17_O_7_PS	Metabolite	301.0504	5.706
Maltol	C_6_H_6_O_3_	Parent	127.0390	4.05
Methomyl	C_5_H_10_N_2_O_2_S	Metabolite	163.0536	5.21
Methoxy-[2-(methylamino)-2-oxoethyl]sulfanylphosphinic acid	C_4_H_10_NO_4_PS	Metabolite	200.0141	0.75
Methoxy-[2-(methylamino)-2-oxoethyl]sulfanylphosphinic acid	C_4_H_10_NO_4_PS	Metabolite	200.0141	1.08
Methyl 2-(difluoromethyl)-4-(2-methylpropyl)-5-(1-oxo-4,5-dihydro-1,3-thiazol-2-yl)-6-(trifluoromethyl)pyridine-3-carboxylate	C_16_H_17_F_5_N_2_O_3_S	Metabolite	413.0953	8.22
Molinate	C_9_H_17_NOS	Parent	188.1104	8.33
Molinate sulfoxide	C_9_H_17_NO_2_S	Metabolite	204.1053	5.81
Molinate sulfoxide	C_9_H_17_NO_2_S	Metabolite	204.1053	5.85
Molinate sulfoxide	C_9_H_17_NO_2_S	Metabolite	204.1053	6.42
Molinate sulfoxide	C_9_H_17_NO_2_S	Metabolite	204.1053	6.85
Molinate sulfoxide	C_9_H_17_NO_2_S	Metabolite	204.1053	6.92
Mono-2-ethyl-5-hydroxyhexyl phthalate	C_16_H_22_O_5_	Metabolite	295.1540	7.57
Mono-8-hydroxyoctyl Phthalate	C_16_H_22_O_5_	Metabolite	295.1540	7.5
mono-Butyl phthalate	C_12_H_14_O_4_	Parent/Metabolite	223.0965	7.67
mono-Methyl phthalate	C_9_H_8_O_4_	Parent	181.0495	6.10
Mono-n-octyl phthalate	C_16_H_22_O_4_	Metabolite	279.1591	8.59
Mono-n-octyl phthalate	C_16_H_22_O_4_	Metabolite	279.1591	8.92
Mono(2-ethyl-5-oxyhexyl)phthalate	C_24_H_38_O_6_	Metabolite	423.2741	5.98
Mono(2E-pentenyl) Phthalate	C_13_H_14_O_4_	Metabolite	235.0965	8.75
Monoisobutyl phthalate	C_12_H_14_O_4_	Metabolite	223.0965	7.65
Monomethyldiuron	C_8_H_8_Cl_2_N_2_O	Metabolite	219.0086	7.49
Monopentyl Phthalate	C_13_H_16_O_4_	Metabolite	237.1121	7.97
Monopentyl Phthalate	C_13_H_16_O_4_	Metabolite	237.1121	8.39
N-(2,4-Dimethylphenyl)formamide	C_9_H_11_NO	Metabolite	150.0913	6.86
N-(3,4-dichloro-2-hydroxyphenyl)acetamide	C_8_H_7_Cl_2_NO_2_	Metabolite	220.9879	7.04
N-(4-Hydroxy-2-methylphenyl)acetamide	C_9_H_11_NO_2_	Metabolite	166.0863	3.35
N-(4-Hydroxyphenyl)-Na(2)-phenylguanidine	C_13_H_13_N_3_O	Metabolite	228.1131	5.15
N-(Methoxyacetyl)glycine	C_5_H_9_NO_4_	Metabolite	148.0604	0.68
N-Ethylaniline	C_8_H_11_N	Parent	122.0964	3.74
N-Ethylcyclohexylamine	C_8_H_17_N	Metabolite	128.1434	3.94
N-Hydroxyurethane	C_3_H_7_NO_3_	Metabolite	106.0499	0.66
N-Methylpyrrolidone	C_5_H_9_NO	Parent	100.0757	3.29
N-Nitrosodiethylamine	C_4_H_10_N_2_O	Parent	103.0866	5.18
n-Propyl 3,4,5-trihydroxybenzoate	C_10_H_12_O_5_	Parent	213.0758	6.37
N,N-Dimethylaniline	C_8_H_11_N	Parent	122.0964	3.34
o-Anisidine	C_7_H_9_NO	Parent	124.0757	2.87
O-Ethyl S-propyl phosphorothioate	C_5_H_13_O_3_PS	Metabolite	185.0396	4.92
O-Ethyl S-propyl phosphorothioate	C_8_H_19_O_3_PS_2_	Metabolite	259.0586	7.12
o-Toluidine	C_7_H_9_N	Parent	108.0808	3.16
Omethoate	C_5_H_12_NO_4_PS	Parent	214.0297	3.95
p-Toluidine	C_7_H_9_N	Parent	108.0808	3.12
Pentaerythritol	C_5_H_12_O_4_	Parent	137.0808	0.73
phenanthro [1,2-b]oxirene-3,5-diol	C_14_H_8_O_3_	Metabolite	225.0546	6.82
phenanthro [1,2-b]oxirene-3,5-diol	C_14_H_8_O_3_	Metabolite	225.0546	7.11
phenanthro [1,2-b]oxirene-3,5-diol	C_14_H_8_O_3_	Metabolite	225.0546	7.35
phenanthro [3,4-b]oxiren-4-ol	C_14_H_8_O_2_	Metabolite	209.0597	7.88
Phenol, 4-((4-cyclopropyl-6-methyl-2-pyrimidinyl)amino)-	C_14_H_15_N_3_O	Metabolite	242.1288	5.97
Phenol, 4,4′-iminobis-	C_12_H_11_NO_2_	Metabolite	202.0863	5.79
Phenylphosphate	C_6_H_7_O_4_P	Metabolite	175.0155	10.44
Phthaldialdehyde	C_8_H_6_O_2_	Parent	135.0441	5.68
Phthalimide	C_8_H_5_NO_2_	Metabolite	148.0393	3.57
Phthalimide	C_8_H_5_NO_2_	Metabolite	148.0393	4.86
Propiconazole	C_15_H_17_Cl_2_N_3_O_2_	Parent	342.0771	8.46
Propiconazole - Mono-hydroxylation/oxidation + Dehydrogenation1	C_15_H_15_Cl_2_N_3_O_3_	Metabolite	356.0559	7.719
Propiconazole_Oxidation_Di-hydroxylation	C_15_H_17_Cl_2_N_3_O_4_	Metabolite	374.0667	6.826
Quinoline-6,8-diol	C_9_H_7_NO_2_	Metabolite	162.0550	3.34
S-(2,3,3-trichloroallyl) (1-hydroxypropan-2-yl)(isopropyl)carbamothioate	C_10_H_16_Cl_3_NO_2_S	Metabolite	320.0040	8.5
S,S-diallyl O-ethyl phosphorodithioate	C_8_H_15_O_2_PS_2_	Metabolite	239.0324	7.21
Sebacic Acid	C_10_H_18_O_4_	Metabolite	203.1278	6.69
Semiamitraz	C_10_H_14_N_2_	Metabolite	163.1230	5.2
Styrene oxide	C_8_H_8_O	Parent	121.0648	7.36
Tebuconazole	C_16_H_22_ClN_3_O	Parent	308.1524	8.33
Thiazopyr	C_16_H_17_F_5_N_2_O_2_S	Parent	397.1004	8.61
Thiazopyr_Oxidation_Dehydrogenation	C_16_H_15_F_5_N_2_O_2_S	Metabolite	395.0843	8.603
Thiazopyr_Oxidation_N/O-Dealkylation/demethylation	C_15_H_15_F_5_N_2_O_2_S	Metabolite	383.0844	7.974
Thioacetamide	C_2_H_5_NS	Parent	76.0215	1.24
Thioacetamide-S-oxide	C_2_H_5_NOS	Metabolite	92.0165	1.14
Thiodicarb	C_10_H_18_N_4_O_4_S_3_	Parent	355.0563	7.37
Tri-allate	C_10_H_16_Cl_3_NOS	Parent	304.0091	9.22
Tributyl phosphate	C_12_H_27_O_4_P	Parent	267.1720	8.66
Tributylamine	C_12_H_27_N	Parent	186.2216	6.29
Tributyltin chloride	C_12_H_27_ClSn	Parent	332.1390	7.72
Triethylene glycol	C_6_H_14_O_4_	Parent	151.0965	2.35
Triethylene glycol dimethyl ether	C_8_H_18_O_4_	Parent	179.1278	5.06
Triethylene glycol dimethyl ether_Oxidation_N/O-Dealkylation/demethylation	C_7_H_16_O_4_	Metabolite	165.1120	4.104
Triglycidyl Isocyanurate	C_12_H_15_N_3_O_6_	Parent	298.1034	5.49
Vanillic Acid	C_8_H_8_O_4_	Metabolite	169.0495	5.22
Vanillin	C_8_H_8_O_3_	Parent	153.0546	5.75

## Data Availability

The raw data supporting the conclusions of this article will be made available by the authors on request.

## Supplementary Materials

The following supporting information can be downloaded at https://www.mdpi.com/article/10.3390/metabo15100650/s1. Table S1: Target Neuroactive Parent Compounds Derived from the ENRICH List; Table S2: Details About the Detectable Parent Compounds and their Metabolites.

## Abbreviations

The following abbreviations are used in this manuscript:

ENRICH	Environmental Neuroactive Chemicals
NTA	Non-targeted analysis
NADPH	Tetrasodium salt
PRM	Parallel-reaction monitoring
AGC	Automatic gain control
MIT	Maximum injection time
DTT	DL-dithiothreitol
*m*/*z*	Mass-to-charge
